# The Incidence of Heart Failure in Children with Congenital Heart Disease: A Prospective Cohort Study

**DOI:** 10.2174/011573403X345783250128052038

**Published:** 2025-02-11

**Authors:** Yasamin Moeinipour, Aliasghar Moeinipour, Behzad Alizadeh, Rasoul Raesi, Mohammadreza Naghibi

**Affiliations:** 1 Cardiothoracic Surgery Division, Cardiothoracic Surgery Department, Faculty of Medicine, Mashhad University of Medical Sciences, Mashhad, Iran;; 2 Pediatric and Congenital Cardiology Division, Pediatric Department, Faculty of Medicine, Mashhad University of Medical Sciences, Mashhad, Iran;; 3 Department of Nursing, Torbat Jam Faculty of Medical Sciences, Torbat Jam, Iran;; 4 Department of Health Services Management, School of Health, Mashhad University *of Medical Sciences, Mashhad, Iran*

**Keywords:** Heart failure (HF), congenital heart disease (CHD), ventricular septal defect (VSD), atrial septal defect (ASD), cardiomyopathy, aortic coarctation

## Abstract

**Introduction:**

Pediatric heart failure (HF) poses diagnostic challenges, especially in emergency settings, where misdiagnoses are common.

**Aim:**

This study aimed to investigate the causes of HF in children with congenital heart disease (CHD) and provide insights into age-related disparities and clinical classifications.

**Methods:**

A prospective observational cohort study was conducted on 402 pediatric patients with CHD during the years 2019-2020. Ultimately, 45 pediatric patients diagnosed with HF by two pediatric cardiologists based on clinical symptoms and radiographic changes were included in the study. Information from the patients' files, including epidemiological findings, clinical examinations, paraclinical findings, and interventions performed, was recorded. Etiological factors and clinical classifications were analyzed using statistical tests.

**Results and Discussion:**

Among 402 pediatric patients with CHD, 45 (11.19%) were diagnosed with HF, with a median age of 7.5 months. The predominant etiological factors included ventricular septal defect (VSD), atrial septal defect (ASD), and cardiomyopathy. Analysis of etiological factors revealed that single structural defects accounted for 71.11% of HF cases, while concurrent defects contributed to a significant portion of the remaining cases. Clinical classifications revealed age-related differences, emphasizing the heterogeneity of pediatric HF presentations.

**Conclusion:**

Given that all patients with HF in our study had CHD, more investigations into the causes and mechanisms of this issue are necessary, which will be possible with genetic studies. A significant difference was observed between Class II and Class IV, with Class II patients being older and heavier, and having a lower heart rate compared to those in Class IV. This aligns with the classifications, where Class II indicates mild symptoms during ordinary activity, while Class IV signifies severe symptoms at rest.

## 1 INTRODUCTION

Heart failure (HF) in children arises from ventricular dysfunction caused by volume or pressure overload or a combination of both [1, 2]. It manifests with distinctive signs and symptoms, including poor growth, feeding difficulties, respiratory distress, exercise intolerance, and fatigue, along with associated abnormalities in circulatory, neurohormonal, and molecular functions [3, 4]. The diagnosis of HF presents a critical medical challenge for pediatric patients. Congenital heart disease (CHD) affects approximately 1–1.2% of live-born infants and is the most common birth defect [5, 6]. CHD encompasses a wide spectrum of structural abnormalities, such as ventricular septal defect (VSD), atrial septal defect (ASD), cardiomyopathy (CM), aortic coarctation (CoA), patent ductus arteriosus (PDA), and aortic stenosis (AS) [6-8]. The chances of survival of a child born with CHD depend on the specific anatomy and level of hemodynamic instability caused by the defects [9, 10]. Recognizing various causes is pivotal for the effective diagnosis and treatment of pediatric cases [11, 12].

The initial presentation of HF in pediatric patients often leads to misdiagnosis of upper respiratory and gastrointestinal infections [5, 13], leading patients to present at emergency departments (ED) with cardiogenic shock [6, 14]. The potential causes differ with age and have an impact on the prognosis; for instance, infants commonly suffer from CHD, whereas acquired heart disease and CMs become more prevalent after the first year of life [15, 16].

CHD varies in terms of complexity, symptoms, and complications. Regrettably, it is also the leading cause of infant mortality associated with congenital anomalies [7]. A recent systematic review demonstrated that primary HF rates in pediatric dilated cardiomyopathy (DCM) patients ranged from 0.87 to 7.4 in one-hundred-thousand population. The study also reported a 40% 5-year mortality rate and/or heart transplant (HT) [17]. Mejia *et al.* (2018) examined 28.6 million pediatric ED visits. About 5971 cases (0.02%) were associated with HF. Notably, these patients were significantly more likely to lead to hospital admission than those without non-HF cases (59.8% *vs*. 4.01%, respectively). During HF-related visits, higher admission rates were observed in patients with CHD and comorbidities. Hospitalized HF patients have a heightened likelihood of mortality. Financially, HF-related ED visits encountered higher costs than non-heart failure visits [18].

Understanding the underlying causes of this condition is imperative to improve patient outcomes. Pediatric HF imposes a significant burden on affected families, both emotionally and financially, owing to frequent interventions. Moreover, It has societal implications, given the associated economic costs. This makes it a pressing public health concern, underscoring the importance of comprehensive investigations [19-21]. Despite remarkable advances in the field of pediatric cardiology, children presenting with HF, particularly in the ED, are diagnostically challenging [22-24].

Timely and accurate diagnosis is critical for improving the prognosis of young patients. Therefore, this study aimed to investigate the causes of HF in children with CHD referred to our medical center. To the best of our knowledge, this is the first study to explore the age at which HF emerges and its specific etiological patterns.

## 2 MATERIALS AND METHODS

### 2.1 Study Design

This prospective cohort study, conducted using a census approach, focused on 402 pediatric patients with CHD to identify and determine the causes of HF in these children. It focused on all pediatric patients under 18 years of age with congenital heart disease (CHD) who were referred to various departments at Imam Reza Hospital in Mashhad, Iran, during the years 2019 and 2020. These departments included the emergency department (ED), neonatal intensive care unit (NICU), pediatric intensive care unit (PICU), and the Pediatric Cardiology Department. Ultimately, out of 402 children with CHD, 45 pediatric patients diagnosed with HF by two pediatric cardiologists, based on clinical symptoms and radiographic findings, were included in the study. The primary outcomes of interest included the age at which HF developed and the various causes of HF, such as congenital, structural, or acquired factors. Additional data collected encompassed demographic information, heart rate, systolic blood pressure, pulmonary artery pressure measured through angiography or echocardiography, duration of hospitalization, specialized care received, paraclinical findings, and radiographic data. Children with CHD were selected based on inclusion and exclusion criteria. Patients who met the inclusion criteria were assessed by two pediatric cardiologists. Ultimately, children diagnosed with HF through clinical examinations and paraclinical diagnostic tests were included in the study.

Inclusion criteria for the study required that all pediatric patients exhibit clinical symptoms suggestive of HF and have a confirmed diagnosis of CHD *via* echocardiography. Patients were excluded if parental consent for participation was not obtained or if they presented with conditions that could mimic HF symptoms, such as infections or sepsis.

### 2.2 Classifications of Pediatric HF

Precise evaluation of the intensity of indications and manifestations of HF in pediatrics poses a considerable challenge due to accurately discerning features from the New York Heart Association (NYHA) classification, such as exertional dyspnea, paroxysmal nocturnal dyspnea (PND), palpitations, neck vein distension, and jugular venous pressure (JVP) [19, 25, 26]. Each participant underwent an assessment based on the modified ROSS classification of CHF by two pediatric cardiologists who were either board-certified or board-eligible (Table **1**).

### 2.3 Data Collection

Data were collected from patient records using a comprehensive checklist including demographic characteristics, vital signs (heart rate and systolic blood pressure), and clinical examinations (dyspnea, electrocardiographic changes, chest X-ray findings, and pulmonary artery pressure measurements obtained from echocardiographic results). Pediatric cardiologists confirmed the diagnosis of HF based on clinical manifestations, electrocardiographic changes, chest X-ray findings, ROSS class, and echocardiographic results. Data were thoroughly obtained from patients' medical records using a standardized checklist, including epidemiological data, clinical evaluations, paraclinical findings, and medical interventions.

### 2.4 Statistical Analysis

Descriptive statistics were used to summarize the data. Statistical tests, including the chi-square test, Mann-Whitney U test, and cross-correlation analysis, were performed to compare means and assess associations between variables. SPSS (version 16) was used with a significance level set at 0.05 for all tests.

## 3 RESULTS

### 3.1 Demographic Characteristics

A total of 402 patients who visited the ED, NICU, PICU, and Pediatric Cardiology departments were included in this study. Fig. (**1**) shows the results of the preliminary analysis of the age distribution of the study participants. The mean age of the participants was 56.8±53.82 months, the median age was 30 months, and the interquartile range (IQR) was 75.63 months. It is apparent from this figure that the age range of the study population varied from newborns to 17 years. Of 402 participants, 196 (48.8%) were male, and 206 (51.2%) were female.

In our study, we identified 45 (11.19%) patients with HF. The median age of the 45 patients was 7.5 months (IQR 23.45). Conversely, individuals with suspected HF (control group= 357 pediatric patients with CHD) who did not receive this diagnosis had a median age of 36 (IQR, 86) months. Statistical analysis using the Mann-Whitney U test revealed a significant age difference between individuals with HF and the control group (*p * 0.0001). The control group included 171 females (47.89%) and 186 males (52.11%), resulting in a gender ratio similar to that of the HF group, which comprised 20 females (44.44%) and 25 males (55.56%). However, there was no significant difference in the gender distribution between those with confirmed CHD and the control group (χ^2^ (1) = 0.938, *p* = 0.33) (Table **2**).

### 3.2 Percentage of Etiological Factors of HF in the Studied Population

Table **3** illustrates some of the etiological factors contributing to the development of HF in 29 cases (64.44%). A single etiological factor, including VSD, ASD, CM, CoA, PDA, and AS, was identified as the primary cause of HF. ASD and VSD occurring concurrently represented the highest percentage (13.33%) of the combined etiological factors leading to HF. ASD and VSD were less frequent, contributing to a smaller proportion of the cases. The co-occurrence of ASD, VSD, and PDA was observed as the etiological factor in 3 patients (6.67%). CM, specifically DCM, was identified as the acquired cause in 6 patients (13.33%). Owing to the distinct nature of these etiological categories, regression models to predict the relationship between CHD and the onset of HF could not be applied. These results provide insight into the etiological factors associated with HF in the study population, emphasizing the importance of recognizing specific etiologies for effective management and intervention strategies.

As shown in Fig. (**2**), a significant difference was observed between classes 2 and 4 in the comparison of age, weight, and heart rate (HR) between the different groups in terms of HF class (*p*0.05). However, there were no significant differences among the other groups. People with class 2 HF had a significantly higher mean age and weight than those with class 4 HF. In addition, people with class 2 HF had a significantly lower average HR per minute than people with HF class 4 (*p*=0.014).

## 4 DISCUSSION

The findings of this study shed light on the causes of HF in children with CHD, emphasizing the importance of early recognition and tailored management strategies. The significant challenges in diagnosing pediatric HF, particularly in the emergency setting, have been highlighted in the introduction, and the study aimed to address these challenges through a comprehensive investigation. In this study, 402 pediatric patients were examined during the period from 2019 to 2020. Ultimately, 45 pediatric patients diagnosed with HF by two pediatric cardiologists based on clinical symptoms and radiographic changes were included in the study. The study revealed that the median age of children diagnosed with HF was 7.5 months, significantly younger than the median age of the overall population screened. This observation aligns with existing literature indicating that HF often presents in infancy, with specific congenital heart defects being predominant in this age group. The diagnostic challenges associated with pediatric HF were underscored, emphasizing the frequent misdiagnosis of upper respiratory and gastrointestinal infections [5]. Recognizing the early signs and implementing screening programs can contribute to timely interventions, potentially improving outcomes for affected children.

The primary etiological factors identified in the study included ventricular septal defect (VSD), atrial septal defect (ASD), cardiomyopathy (CM), aortic coarctation (CoA), patent ductus arteriosus (PDA), and aortic stenosis (AS). The co-occurrence of multiple defects, such as ASD and VSD, highlighted the complex nature of CHD and its potential impact on the development of HF. Hirono *et al.* examined 53 Japanese children with Left Ventricular Noncompaction and CHD. They concluded that 31 (58.5%) patients experienced HF. This study concurs with the fact that a high percentage of CHD experience HF [27]. Among the patients examined, 29 cases (54.7%) had ventricular wall defects (VSD), 17 cases (32.1%) had atrial wall defects (ASD), 10 cases (18.8%) had PDA, and 7 cases (13.2%) had Ebstein anomaly and double outlet right ventricle. Our study showed a smaller percentage of subjects with VSD or ASD than their study, which may be due to genetic differences in the studied populations. Our study did not include a genetic analysis.

Comprehensive epidemiological studies on HF in the pediatric population are limited. However, several studies in Europe have shown that more than half of the cases of HF in children are related to CHD [28, 29]. In a study by Massin *et al.*, 1,196 children with CHD were examined over 10 years. Of 124 cases of HF among these patients (10.4%), 72 cases (58.1%) developed HF in the first year of life. In our study, out of 45 people with HF, 21 people (46.6%) had it during the first year of life, which aligns with the study of Massin *et al.* (58.1%) [12].

The study utilized modified ROSS classifications to assess the severity of HF. The observed differences between classes 2 and 4 in terms of age, weight, and heart rate provide valuable insights into the clinical presentation and progression of pediatric HF. These distinctions underscore the heterogeneous nature of HF in children, necessitating personalized approaches to care. Unlike our study, Norozi *et al.* reported that in 345 patients with CHD, the HF criteria were confirmed in 89 patients. These patients were significantly older than others (mean 30.8) [30]. However, this difference arose from the age group under investigation. The patients in our study were all under 18 years of age, while Norozi *et al.* aimed to investigate the role of CHD in the incidence of HF in adults. The findings of the study have practical implications for developing screening programs tailored to the specific etiological patterns observed. Early identification of congenital heart defects, especially in high-risk populations, can guide preventive measures and targeted interventions. The prevalence of CHD in pediatric HF cases reinforces the need for ongoing efforts to enhance prenatal and neonatal screening programs, ensuring early detection and timely management. Also, Speckle-tracking echocardiography is increasingly recognized as a vital tool for the early detection of subclinical myocardial dysfunction in the pediatric population [31]. This advanced echocardiographic technique allows for the detailed assessment of myocardial deformation properties, specifically measuring strain and strain rate in both ventricles and atria [32]. Unlike conventional echocardiography, which primarily evaluates global and regional wall motion, speckle-tracking echocardiography provides a more sensitive and comprehensive analysis of biventricular systolic function [33]. Research has shown that this technique can identify subtle changes in myocardial mechanics before overt clinical symptoms arise, making it particularly valuable for monitoring children at risk for cardiac dysfunction due to congenital heart disease or other underlying conditions [33, 34]. The implementation of speckle-tracking echocardiography into routine clinical practice can facilitate earlier intervention and tailored management strategies, ultimately leading to improved outcomes for pediatric patients with cardiovascular issues [35, 36].

## CONCLUSION

This study contributes valuable information regarding the age at which heart failure manifests and the specific etiological patterns in children with CHD. The identified causes, prevalence of CHD, and clinical classifications offer a foundation for refining diagnostic and intervention strategies. The implications for screening programs, early detection, and personalized management underscore the importance of a multidisciplinary approach in pediatric cardiology. Further research and collaborative efforts are warranted to advance our understanding of pediatric heart failure and improve outcomes for affected children.

## LIMITATIONS OF THE STUDY

While the study provides valuable insights, it is essential to acknowledge its limitations. The single-center nature of the study and the relatively small sample size may limit the generalizability of the findings. The most significant limitation was the lack of investigation into other causes of heart disease and rhythm disorders. The exclusion criteria for non-obtained parental consent and the exclusion of patients with conditions mimicking HF symptoms could have influenced the results. Future research endeavors could involve multi-center collaborations to enhance the robustness and applicability of the results. Additionally, long-term follow-up studies could offer insights into the outcomes and survival rates of children diagnosed with HF in early infancy.

## Figures and Tables

**Fig. (1):**
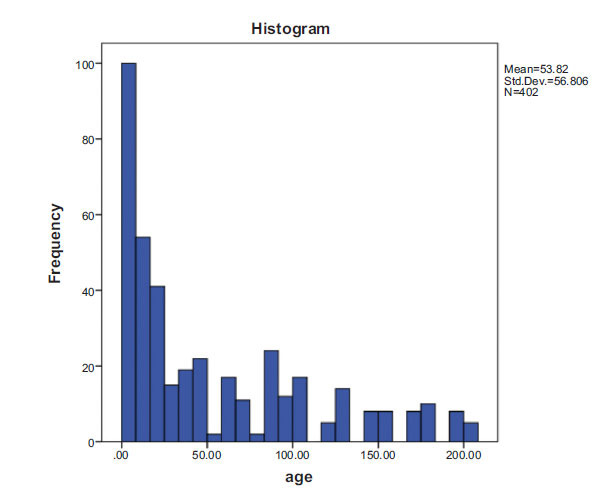
Age distribution of examined patients by m.

**Fig. (2):**
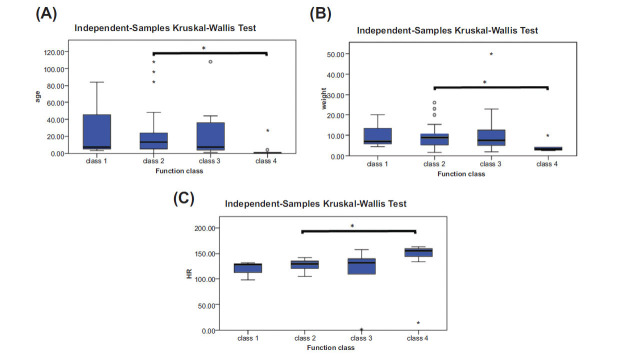
The comparison of age, weight, and heart rate (HR) between the different groups in terms of HF class. (**A**): Age comparison (months); (**B**): Weight comparison (kilogram); (**C**): Comparison of heart rate (per minute) between different functional classes of heart failure. Asterisks (*) denote outliers.

**Table 1:** The comparison of the New York Heart Association (NYHA) and ROSS classification (11-13).

**-**	**NYHA**	**ROSS Classification**
Class I	Symptom onset with more than ordinary level of activity	Asymptomatic
Class II	Symptom onset with an ordinary level of activity	Mild tachypnea or diaphoresis with feeding in infants
Class II	Symptom onset with minimal activity	Marked tachypnea or diaphoresis with feeding in infants and prolonged feeding times with growth failure; marked dyspnea on exertion in older children
Class IV	Symptoms of heart failure at rest. Any physical activity causes further discomfort	Tachypnea, retractions, grunting, or diaphoresis at rest

**Table 2:** Demographic characteristics of the studied population.

**-**		**HF**		**Control Group**		*p* Value*
		**Frequency**	**P (%)**	**Frequency**	**P (%)**	
Gender	Female	20	44.44	171	47.89	0.33
	Male	25	55.56	186	52.11	

*Chi-square test

*p Value 0.05

**Table 3:** Etiological factors of heart failure in studied populations.

**Etiology of Heart Failure**	**Frequency**	**Percentage (%)**
VSD	4	8.89
ASD	1	2.22
Cardiomyopathy	6	13.33
Aortic coarctation	6	13.33
PDA	6	13.33
AS	6	13.33
ASD, VSD, and PDA	3	6.7
ASD and VSD	6	13.33
ASD and PDA	2	4.44
AS and VSD	1	2.22
TAPVC	3	6.7
TAPVC and aorta coarctation	1	2.2

**Abbreviations:** VSD: ventricular septal defect; ASD: atrial septal defect; PDA: patent ductus arteriosus; AS: aortic stenosis; TAPVC: Total anomalous pulmonary venous connection.

## Data Availability

The data that support the findings of this study are available from the corresponding author upon reasonable request.

## LIST OF ABBREVIATIONS

ASD: Atrial Septal Defect
CHD: Congenital Heart Disease
HF: Heart Failure
NYHA: New York Heart Association
PND: Paroxysmal Nocturnal Dyspnea
VSD: Ventricular Septal Defect

## STANDARDS OF REPORTING

STROBE guidelines were followed.

## References

[1] Kantor P.F., Abraham J.R., Dipchand A.I., Benson L.N., Redington A.N. (2010). The impact of changing medical therapy on transplantation-free survival in pediatric dilated cardiomyopathy.. J. Am. Coll. Cardiol..

[2] Macicek S.M., Macias C.G., Jefferies J.L., Kim J.J., Price J.F. (2009). Acute heart failure syndromes in the pediatric emergency department.. Pediatrics.

[3] Masarone D., Valente F., Rubino M., Vastarella R., Gravino R., Rea A., Russo M.G., Pacileo G., Limongelli G. (2017). Pediatric heart failure: A practical guide to diagnosis and management.. Pediatr. Neonatol..

[4] Das B.B. (2018). Current state of pediatric heart failure.. Children.

[5] Hollander S.A., Addonizio L.J., Chin C., Lamour J.M., Hsu D.T., Bernstein D., Rosenthal D.N. (2013). Abdominal complaints as a common first presentation of heart failure in adolescents with dilated cardiomyopathy.. Am. J. Emerg. Med..

[6] Watanabe K., Shih R. (2020). Update of pediatric heart failure.. Pediatr. Clin. North Am..

[7] van der Bom T., Zomer A.C., Zwinderman A.H., Meijboom F.J., Bouma B.J., Mulder B.J.M. (2011). The changing epidemiology of congenital heart disease.. Nat. Rev. Cardiol..

[8] Hoch M, Netz H (2005). Heart failure in pediatric patients.. J Thorac. Cardiovasc. Surg..

[9] Rossano J.W., Jang G.Y. (2015). Pediatric heart failure: Current state and future possibilities.. Korean Circ. J..

[10] Auslender M. (2000). Pathophysiology of pediatric heart failure.. Prog. Pediatr. Cardiol..

[11] Connolly D., Rutkowski M., Auslender M., Artman M. (2001). The New York University pediatric heart failure index: A new method of quantifying chronic heart failure severity in children.. J. Pediatr..

[12] Massin M.M., Astadicko I., Dessy H. (2008). Epidemiology of heart failure in a tertiary pediatric center.. Clin. Cardiol..

[13] Auerbach S.R., Richmond M.E., Lamour J.M., Blume E.D., Addonizio L.J., Shaddy R.E., Mahony L., Pahl E., Hsu D.T. (2010). BNP levels predict outcome in pediatric heart failure patients: Post hoc analysis of the Pediatric Carvedilol Trial.. Circ. Heart Fail..

[14] Auslender M., Artman M. (2000). Overview of the management of pediatric heart failure.. Prog. Pediatr. Cardiol..

[15] Kirk R., Dipchand A.I., Rosenthal D.N., Addonizio L., Burch M., Chrisant M., Dubin A., Everitt M., Gajarski R., Mertens L., Miyamoto S., Morales D., Pahl E., Shaddy R., Towbin J., Weintraub R. (2014). The International Society for heart and lung transplantation guidelines for the management of pediatric heart failure: Executive summary.. J. Heart Lung Transplant..

[16] Wilkinson J.D., Landy D.C., Colan S.D., Towbin J.A., Sleeper L.A., Orav E.J., Cox G.F., Canter C.E., Hsu D.T., Webber S.A., Lipshultz S.E. (2010). The pediatric cardiomyopathy registry and heart failure: Key results from the first 15 years.. Heart Fail. Clin..

[17] Shaddy R.E., George A.T., Jaecklin T., Lochlainn E.N., Thakur L., Agrawal R., Solar-Yohay S., Chen F., Rossano J.W., Severin T., Burch M. (2018). Systematic literature review on the incidence and prevalence of heart failure in children and adolescents.. Pediatr. Cardiol..

[18] Mejia E.J., O'Connor M.J., Lin K.Y., Song L., Griffis H., Mascio C.E., Shamszad P., Donoghue A., Ravishankar C., Shaddy R.E., Rossano J.W. (2018). Characteristics and outcomes of pediatric heart failure-related emergency Department Visits in the United States: A Population-Based study.. J. Pediatr..

[19] Hsu D.T., Pearson G.D. (2009). Heart failure in children: Part I: history, etiology, and pathophysiology.. Circ. Heart Fail..

[20] Ohuchi H., Takasugi H., Ohashi H., Okada Y., Yamada O., Ono Y., Yagihara T., Echigo S. (2003). Stratification of pediatric heart failure on the basis of neurohormonal and cardiac autonomic nervous activities in patients with congenital heart disease.. Circulation.

[21] Jefferies J.L., Denfield S.W., Price J.F., Dreyer W.J., McMahon C.J., Grenier M.A., Kim J.J., Dimas V.V., Clunie S.K., Moffett B.S., Chang A.C., Wann T.I., Smith E.O., Towbin J.A. (2006). A prospective evaluation of nesiritide in the treatment of pediatric heart failure.. Pediatr. Cardiol..

[22] Šamánek M. (1992). Children with congenital heart disease: Probability of natural survival.. Pediatr. Cardiol..

[23] Lewis K.D., Conway J., Cunningham C., Larsen B.M.K. (2018). Optimizing nutrition in pediatric heart failure: The crisis is over and now it’s time to feed.. Nutr. Clin. Pract..

[24] Yuerek M., Rossano J.W., Mascio C.E., Shaddy R.E. (2016). Postoperative management of heart failure in pediatric patients.. Expert Rev. Cardiovasc. Ther..

[25] Ross R.D. (2012). The Ross classification for heart failure in children after 25 years: A review and an age-stratified revision.. Pediatr. Cardiol..

[26] Ross R.D., Bollinger R.O., Pinsky W.W. (1992). Grading the severity of congestive heart failure in infants.. Pediatr. Cardiol..

[27] Hirono K., Hata Y., Miyao N., Okabe M., Takarada S., Nakaoka H., Ibuki K., Ozawa S., Yoshimura N., Nishida N., Ichida F. (2020). Left ventricular noncompaction and congenital heart disease increases the risk of congestive heart failure.. J. Clin. Med..

[28] Gilljam T., Mandalenakis Z., Dellborg M., Lappas G., Eriksson P., Skoglund K., Rosengren A. (2019). Development of heart failure in young patients with congenital heart disease: A nation-wide cohort study.. Open Heart.

[29] Hinton R.B., Ware S.M. (2017). Heart failure in pediatric patients with congenital heart disease.. Circ. Res..

[30] Norozi K., Wessel A., Alpers V., Arnhold J.O., Geyer S., Zoege M., Buchhorn R. (2006). Incidence and risk distribution of heart failure in adolescents and adults with congenital heart disease after cardiac surgery.. Am. J. Cardiol..

[31] Forsey J., Friedberg M.K., Mertens L. (2013). Speckle tracking echocardiography in pediatric and congenital heart disease.. Echocardiography.

[32] Dorobantu D.M., Wadey C.A., Amir N.H., Stuart A.G., Williams C.A., Pieles G.E. (2021). The role of speckle tracking echocardiography in the evaluation of common inherited cardiomyopathies in children and adolescents: A systematic review.. Diagnostics.

[33] Dorobantu D.M., Amir N.H., Wadey C.A., Sharma C., Stuart A.G., Williams C.A., Pieles G.E. (2024). The role of speckle-tracking echocardiography in predicting mortality and morbidity in patients with congenital heart disease: A systematic review and meta-analysis.. J. Am. Soc. Echocardiogr..

[34] Chan J.C., Menon A.P., Rotta A.T., Choo J.T.L., Hornik C.P., Lee J.H. (2024). Use of speckle-tracking echocardiography in septic cardiomyopathy in critically Ill children: A narrative review.. Crit. Care Explor..

[35] Mohammad Nijres B., Bokowski J., Al-Kubaisi M., Abdulla R., Murphy J.J., Awad S., Diab K.A. (2018). Use of speckle tracking echocardiography to assess left ventricular systolic function in patients with surgically repaired tetralogy of Fallot: Global and segmental assessment.. Pediatr. Cardiol..

[36] Luis S.A., Chan J., Pellikka P.A. (2019). Echocardiographic assessment of left ventricular systolic function: An overview of contemporary techniques, including speckle-tracking echocardiography.. Mayo. Clin. Proc..

